# Evaluation of *Streptococcus mutans* biofilm formation on fluoride‐releasing materials used as pit and fissure sealants

**DOI:** 10.1111/eos.70034

**Published:** 2025-07-28

**Authors:** Patrícia da S. L. Pereira da Silva, Mariana da Silva Teixeira, Anderson de Araújo Rocha, Rosana Rocha Barros, Lucio Sousa Gonçalves, Mônica Almeida Tostes

**Affiliations:** ^1^ School of Dentistry Fluminense Federal University Niterói Rio de Janeiro Brazil; ^2^ Biomass Study and Water Management Center Fluminense Federal University Niterói Rio de Janeiro Brazil; ^3^ Department of Microbiology and Parasitology Fluminense Federal University Niterói Rio de Janeiro Brazil; ^4^ School of Dentistry Estácio de Sá University Rio de Janeiro Rio de Janeiro Brazil

**Keywords:** bacterial adhesion, caries dental, dental materials, fluoride treatment, *Streptococcus mutans*

## Abstract

This in vitro study evaluated fluoride release and *Streptococcus mutans* biofilm formation on four types of dental sealants: a giomer, resin‐modified glass ionomer, compomer, and a fluoride‐free composite (control). Discs were prepared as per the manufacturers’ instructions, and they were placed in tubes containing ultrapure water. Fluoride release was measured after 2, 7, and 30 days using ion chromatography, and biofilm formation was analyzed via scanning electron microscopy (SEM). A repeated‐measures linear mixed model with log transformation and Bonferroni‐corrected post‐hoc tests was applied. The resin‐modified glass ionomer showed the highest fluoride release, followed by the giomer, with increasing values over time. Compomer and the fluoride‐free composite exhibited low, stable fluoride levels. SEM confirmed *S. mutans* biofilm on all materials. These findings highlight distinct fluoride release profiles and suggest that materials with higher fluoride release may offer improved long‐term protection against cariogenic biofilm formation.

## INTRODUCTION

The effectiveness of fluoride‐containing materials in caries prevention has led to their widespread use in pediatric dentistry as minimally invasive interventions [1, 2, 3]. Dental caries result from an imbalance between cariogenic biofilm formation, fueled by fermentable carbohydrate consumption, and protective factors such as salivary flow, biofilm control, and fluoride exposure [2, 4, 5].

Since the mid‐1960s, pit and fissure sealants have been employed as physical barriers to biofilm accumulation, with some materials also providing fluoride release as a chemical defense [6]. Evidence supports their role in reducing caries incidence on occlusal surfaces of primary and permanent molars, outperforming the absence of sealants or fluoride varnish applications [7]. The application of sealants to the pits and fissures is a reliable and effective strategy for both caries prevention and arrest. However, sustained effectiveness relies on periodic dental evaluations and reapplication whenever necessary [8]. Recent advancements in biomaterials have introduced antimicrobial and bioactive properties to enhance sealant efficacy, either by improving physical barriers or releasing components that modulate biofilm formation [4, 9]. Notably, fluoride release serves as a key characteristic of these materials, influencing biofilm dynamics by interfering with salivary pellicle formation, reducing bacterial adhesion, and inhibiting microbial growth and metabolism within the biofilm [10, 11, 12]. These combined effects contribute to limiting cariogenic biofilm development and enhancing the overall preventive capacity of sealants [13, 14]. *Streptococcus mutans* is a key contributor to cariogenic biofilms due to its capacity to metabolize carbohydrates, produce acids that demineralize enamel, and form an extracellular matrix that facilitates bacterial adhesion and retention [15]. Its adaptation to acidic conditions gives it a competitive advantage in colonizing tooth surfaces [16]. Given its leading role in biofilm formation and caries development, strategies targeting *S. mutans* are essential for effective caries prevention [17].

To address the need for fluoride‐releasing materials that effectively inhibit biofilm formation, this study evaluated three fluoride‐containing sealants: giomer, a resin‐based material incorporating surface pre‐reacted glass‐ionomer (S‐PRG) technology; a light‐cured, reinforced glass ionomer cement, which offers improved physical properties and reduced moisture sensitivity; and a compomer combining resin‐based polymerization with fluoride release. A fluoride‐free composite served as the negative control [7, 18, 19, 20]. Therefore, this in vitro study aimed to quantify fluoride release and to evaluate the formation of *S. mutans* ATCC 25,175 biofilm on the surface of these sealants, providing insights into their potential efficacy in caries prevention and microbial control.

## MATERIAL AND METHODS

### Specimen preparation

The assessed dental sealants are described in Table 1. All materials are recommended for pit and fissure sealing. Initially, 12 discs of each sealant, measuring 5 mm in diameter and 3 mm in thickness, were prepared using metal molds covered with acetate [21, 22]. These discs were light‐cured in accordance with the manufacturers' instructions. The discs were examined under a 10× objective optical microscope, and one with visible surface alterations (e.g., bubbles) was excluded in each material group. The discs were washed and stored in a moist container at 37°C for 1 h [23]. Nine specimens were assigned for quantitative fluoride analysis, while two were reserved for evaluation of bacterial adhesion using scanning electron microscopy (SEM).

**TABLE 1:** Description of the tested dental materials.

Dental material	Brand name/manufacturer	Composition	Type
Giomer	BeautiSealant, Shofu	Technologies s‐PRG particles, free from BPA and HEMA	Hybrid
Resin‐modified glass ionomer	Riva Light Cure, SDI	Compartment 1: polyacrylic acid, tartaric acid, HEMA, EDGMA, acid monomers; Compartment 2: fluoride and aluminum silicate glass	Glass ionomer
Compomer	Twinky Star Flow, VOCO	65% inorganic fill, methacrylates, BisGMA, UDMA, TEGMA	Hybrid
Fluoride‐free composite	Sigma Flow, DFL	Dimethacrylate, BisGMA, Boron aluminum silicate glass, synthetic silica, and pigments	Composite

Abbreviations: BisGMA, bisphenol A glycidyl methacrylate; BPA, bisphenol A; EDGMA, ethylene glycol dimethacrylate; HEMA, 2‐hydroxymethyl methacrylate; sPRG, surface pre‐reacted glass‐ionomer; TEGMA, triethylene glycol dimethacrylate; UDMA, urethane dimethacrylate.

The sample size for fluoride release was defined based on a review of previous in vitro studies evaluating fluoride release from restorative dental materials, including pit and fissure sealants [13, 23, 24, 25]. According to these references, most studies adopt between 6 and 10 specimens per group, which has been shown to be adequate to detect moderate‐to‐large differences in fluoride release using one‐way ANOVA under a significance level of 5% (*α* = 0.05) and statistical power of 80% (1 − *β* = 0.80).

For the qualitative and quantitative analyses of bacterial adhesion by SEM, two specimens per type of sealant (*n* = 2) were analyzed using SEM following exposure to an *S. mutans* (ATCC25175) inoculum of 10^6^ CFU/mL at 37°C for 48 h. The sample size for this part of the study was established based on the exploratory nature of the analysis and its primarily visual and morphological purpose. The SEM assessments were performed under standardized magnification and preparation conditions to allow representative comparisons of surface colonization patterns between materials. Previous literature supports the use of small sample sizes (*n* = 2–3) for SEM in similar in vitro models, as the technique is not used to generate inferential statistics but rather to confirm and illustrate trends identified through quantitative methods [10, 11]. In this context, the second part of the study evaluated the presence and quantified the density of *S. mutans* biofilm structures on the surface of the tested dental sealants under standardized biofilm growth conditions.

### Fluoride release assay and quantification by ion chromatography

Fluoride release was assessed in triplicate by placing the specimens in Falcon tubes (Sarstedt) containing ultrapure water (resistivity > 18 Mohm), obtained from the Integral 5 water purifier device (Millipore). The release assay started after homogenization of the medium. The specimens were incubated in an oven (Ssa‐40L, 7Lab) at 37°C, and aliquots were withdrawn after 2, 7, and 30 days. For each sampling, 3 mL of solution was extracted using a syringe and subsequently filtered through a 0.45 µm cellulose acetate membrane.

Fluoride release into the solution was monitored over time, using ion chromatography (850 Professional IC, Metrohm) for analysis of the analyte. The quantification of fluoride release was measured in µg/cm^2^ and converted to parts per million (ppm). An analytical fluoride curve was built within the range of 0.1 to 5.0 ppm, based on a 1000 ppm fluoride standard (Fluka Analytical, Primus). Both the standards for the analytical curve and the filtered specimens were loaded into an automated sampler (858 Professional Sample Processor, Metrohm) and subsequently transferred sequentially into a 20 µL  loop. They were then eluted using a mobile phase consisting of a mixture of 2.0 mM sodium bicarbonate (Merck) and 3.0 mM sodium hydroxide (Merck), at a constant elution rate of 0.8 mL·min^−1^. The stationary phase consisted of an anion separation column (MetroSep A Sup 5150 × 4 mm, Metrohm), featuring polyvinyl alcohol packing with quaternary ammonium groups and a particle size of 5 mm.

**FIGURE 1:**
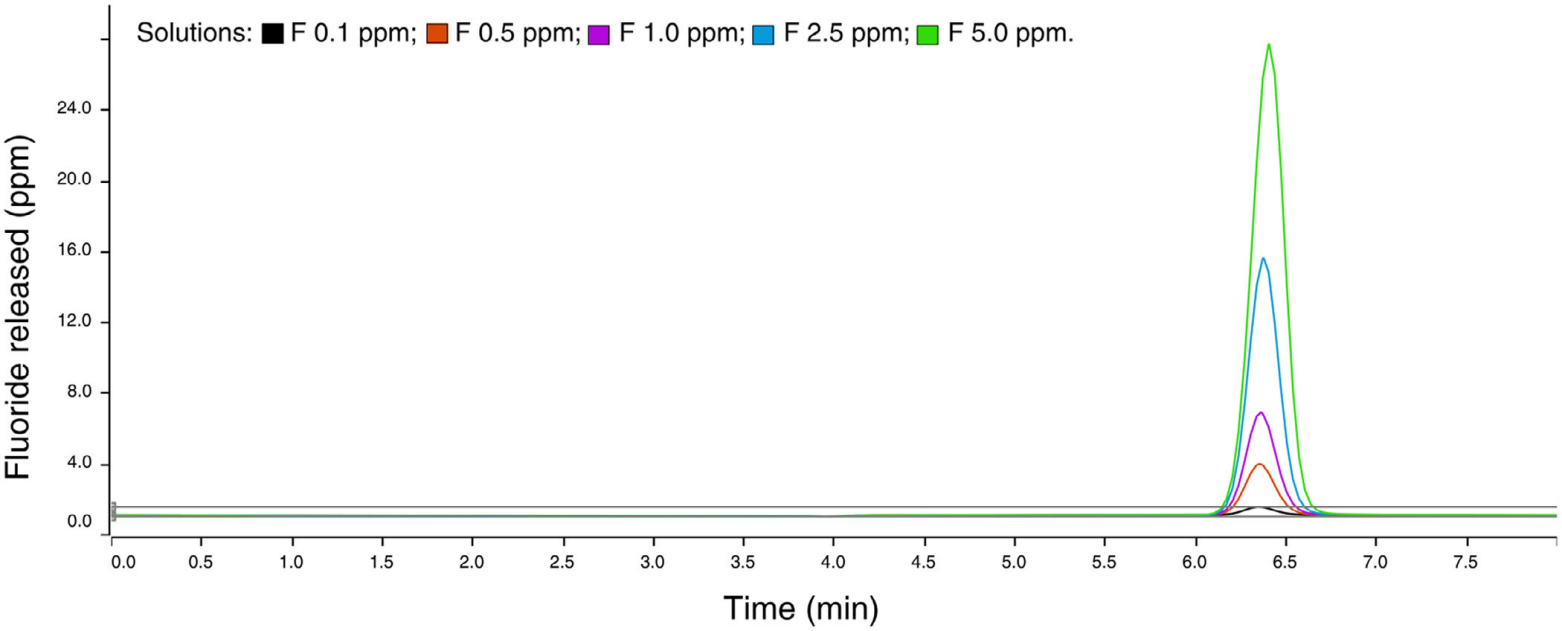
Analytical curve showing the peak corresponding to the fluoride ion released.

Detection was carried out using a conductimetric system. An ionization suppression system was employed, incorporating 5% H_2_SO_4_ (Merck, Sigma‐Aldrich) and ultrapure water, to suppress the conductivity of the eluent. The results were interpreted utilizing the magic net 3.2 software provided with the instrument, which identified the peak for the fluoride ion at 6.4 min. The analyte concentration in the specimens was quantified using the equation derived from the linear regression (*r* > 0.999) of the analytical curve, considering the peak height (Figure 1) [26].

### Analysis of biofilm production and adhesion using scanning electron microscopy

Scanning electron microscopy plays a key role in the qualitative analysis of biofilm formation and adhesion on sealant surfaces, allowing for the detection of potential surface irregularities, morphology, and density of adherent elements [27]. The sealant discs (*n* = 2/ type of sealant) were exposed to an *S. mutans* (ATCC25175) inoculum of 10^6^ CFU/mL, in brain heart infusion (BHI) medium enriched with 2% sucrose (Becton Dickinson), incubated under microaerophilic conditions at 37°C for 48 h. The sealants were then treated with 2.5% glutaraldehyde (Sigma‐Aldrich) for 1 h, followed by immersion in 0.1 M sodium cacodylate buffer, and three 15‐min washes with the buffer. After initial preparation, the sealant discs were dehydrated with acetone using a stepwise protocol, consisting of 15‐min immersions at concentrations of 30%, 50%, 70%, 90%, and 100%. The discs were kept in the 100% acetone solution for three additional 15‐min cycles. After drying, the specimens were mounted onto metal stubs and gold‐sputtered using the Ion Sputter Coater G20 system (S3 Alliance) and imaged using a scanning electron microscope (JEOL JSM‐7100F Field Emission SEM) at 10,000× magnification before and after 48 h of *S. mutans* biofilm growth [28].

In addition to the qualitative analysis, the SEM images were quantitatively evaluated to assess *S. mutans* adhesion. This analysis was conducted using Fiji, an open‐source distribution of ImageJ widely utilized in biomedical imaging [29]. Image segmentation was performed with the Trainable Weka Segmentation plugin, a supervised machine learning tool that classifies pixels based on extracted features and training with manually annotated regions [30]. Importantly, this evaluation was carried out in a double‐blind manner to ensure objectivity and to reduce bias.

### Statistical analysis

Statistical analyses were performed to assess fluoride release from four distinct restorative materials at three experimental time points (2, 7, and 30 days). All analyses were conducted using R (version 4.4.3) and Jamovi (version 2.6.44). The significance level was set at *α* = 0.05 [31, 32, 33].

As an initial step, descriptive statistics were generated for the observed amount of fluoride released, including means, standard errors, and 95% confidence intervals for each combination of material and assessment period. Visual inspection of the data showed different patterns of fluoride release over time among the materials, indicating the need for models that included the interaction between time and material.

Considering the study design, which involved within‐individual replications and two fixed factors (material and time), a linear mixed model for the inferential analyses was employed. This model was chosen because of its robustness in handling hierarchical data structures and repeated measures and its ability to tackle potential imbalances, violations of sphericity, and heterogeneity of variance across groups [31].

To meet the assumptions of parametric tests, data were initially assessed for normality and sphericity. In the presence of skewness or heteroscedasticity in the residuals, a logarithmic transformation (log₁₀) was applied to the raw values to stabilize variance, reduce the influence of outliers, and approximate a normal distribution, thereby supporting the use of linear models. Normality was evaluated using the Shapiro–Wilk and Kolmogorov–Smirnov tests on the standardized residuals. Violation of this assumption justified the transformation, after which the model assumptions were satisfactorily met [32, 33, 34, 35].

The procedure also helped to fulfill the sphericity criterion in repeated measures designs, ensuring the validity of subsequent inferences. All results were interpreted with caution, considering the transformed scale. It is important to note that while the logarithmic transformation effectively corrects residual asymmetry, it may amplify minor variations in materials with minimal or no fluoride release [32, 33, 34]. Consequently, findings related to the fluoride‐free composite should be interpreted cautiously, as statistically significant differences may not correspond to clinically relevant outcomes.

Using the log‐transformed variable, the linear mixed model was fitted with a random intercept for the “individual” factor, incorporating within‐individual variabilities as a random effect. The fixed factors in the model were time, material, and their interaction. The significance of the effects was assessed using F tests derived from the fitted model. The detection of a significant interaction between time and material supported the application of post‐hoc tests [31].

Multiple comparisons were conducted based on the linear mixed model using pairwise comparisons with Bonferroni‐corrected *p*‐values. This was done for comparisons between time points for each material (within‐group comparison) and for comparisons between materials at each time point (between‐group analysis). The differences were initially derived from the logarithmic scale and subsequently exponentiated (exp [Difference]) for their return to the original scale, enabling the practical interpretation of the results in terms of fluoride release.

Generalized effect sizes (η^2^g) were calculated to assess the magnitude of the observed effects based on a repeated measures linear mixed model using the rstatix package in R. The database was organized in long format, incorporating time (within‐individual), material (between‐individual), and id (individual). Effect sizes were interpreted according to the recommended reference values: η^2^g ≈ 0.01 (small), 0.06 (moderate), and ≥ 0.14 (large) [31, 32, 33, 34, 35, 36]. The calculated values were reported for the main effects of time and material, as well as for their interaction, and used to qualify the statistical significance of the findings. These effect sizes provide a quantitative estimate of how much fluoride release was influenced by each factor, indicating the proportion of variance in fluoride release explained by time, material type, and their interaction. Higher values reflect stronger associations, thereby supporting the scientific relevance of the results.

## RESULTS

The results of the statistical analysis are presented in Tables 2 and 3, which include the significance test outcomes for the main effects of time and material, as well as their interaction, on fluoride release (dependent variable), suggesting that the temporal pattern of fluoride release differed depending on the material, that is, the materials responded differently to the time factor. All effects exhibited high generalized effect sizes (η^2^g), implying that the examined factors accounted for a substantial portion of the variability in fluoride release.

**TABLE 2:** Post‐hoc comparisons between time points within each material for fluoride release in ppm (logarithmic and original scales).

Comparison							
Material	Day		Material	Day	Difference fluoride release (log‐scale)	Exponentiated log difference (ppm)	Bonferroni corrected *p*‐value
Compomer	2	vs.	Compomer	7	0.003	1.00	ns
Compomer	2	vs.	Compomer	30	0.055	1.06	ns
Compomer	7	vs.	Compomer	30	0.052	1.05	ns
Fluoride‐free composite	2	vs.	Fluoride‐free composite	7	−0.091	0.91	ns
Fluoride‐free composite	2	vs.	Fluoride‐free composite	30	−0.319	0.73	0.029
Fluoride‐free composite	7	vs.	Fluoride‐free composite	30	−0.228	0.80	0.415
Giomer	2	vs.	Giomer	7	−0.252	0.78	0.206
Giomer	2	vs.	Giomer	30	−0.795	0.45	<0.001
Giomer	7	vs.	Giomer	30	−0.543	0.58	<0.001
Reinforced glass ionomer cement	2	vs.	Reinforced glass ionomer cement	7	−0.507	0.60	<0.001
Reinforced glass ionomer cement	2	vs.	Reinforced glass ionomer cement	30	≈ ‐inf	≈ 0	<0.001
Reinforced glass ionomer cement	7	vs.	Reinforced glass ionomer cement	30	−0.636	0.53	<0.001

*Note*: Logarithmic differences approaching –inf indicate that the reference group exhibited near‐zero fluoride release, resulting in an exponential ratio close to 0 on the original scale.

**TABLE 3:** Post‐hoc comparisons between materials within each time point for fluoride release in ppm (logarithmic and original scales).

Comparison							
Material	Day		Material	Day	Difference (log‐scale)	Exponentiated log difference (ppm)	Bonferroni corrected *p*‐value
Compomer	2	vs.	Fluoride‐free composite	2	0.218	1.24	ns.
Compomer	2	vs.	Giomer	2	−0.749	0.47	<0.001
Compomer	2	vs.	Reinforced glass ionomer cement	2	−0.653	0.52	<0.001
Compomer	7	vs.	Fluoride‐free composite	7	0.124	1.13	ns.
Compomer	7	vs.	Giomer	7	−100	≈ 0	<0.001
Compomer	7	vs.	Reinforced glass ionomer cement	7	−116	≈ 0	<0.001
Compomer	30	vs.	Fluoride‐free composite	30	−0.156	0.86	ns.
Compomer	30	vs.	Giomer	30	−160	≈ 0	<0.001
Compomer	30	vs.	Reinforced glass ionomer cement	30	−185	≈ 0	<0.001
Fluoride‐free composite	2	vs.	Giomer	2	−0.967	0.38	<0.001
Fluoride‐free composite	2	vs.	Reinforced glass ionomer cement	2	−0.872	0.42	<0.001
Fluoride‐free composite	7	vs.	Giomer	7	−113	≈ 0	<0.001
Fluoride‐free composite	7	vs.	Reinforced glass ionomer cement	7	−129	≈ 0	<0.001
Fluoride‐free composite	30	vs.	Giomer	30	−144	≈ 0	<0.001
Fluoride‐free composite	30	vs.	Reinforced glass ionomer cement	30	−170	≈ 0	<0.001
Giomer	2	vs.	Reinforced glass ionomer cement	2	0.096	1.1	ns.
Giomer	7	vs.	Reinforced glass ionomer cement	7	−0.160	0.85	ns.
Giomer	30	vs.	Reinforced glass ionomer cement	30	−0.253	0.78	0.411

Fluoride release data were calculated for each material and time point combination (Tables 2 and 3), with 95% confidence intervals for the mean, based on three specimens per time point (*n* = 9 per group). A notable change in fluoride values was observed between days 2 and 30 for the fluoride‐free composite. However, this variation should be interpreted cautiously, as the material does not contain fluoride in its composition. Minor fluctuations in measurements, especially those near or below the detection threshold, may be amplified by logarithmic transformation, without practical implications. Given the sample size of *n* = 9 per group, these standard errors may be unstable, and the confidence intervals may include negative values. While these values lack practical meaning, they are statistically possible. The results derived from the adjusted model, based on transformed data with verified assumptions, are presented in Tables 2 and 3.

**FIGURE 2:**
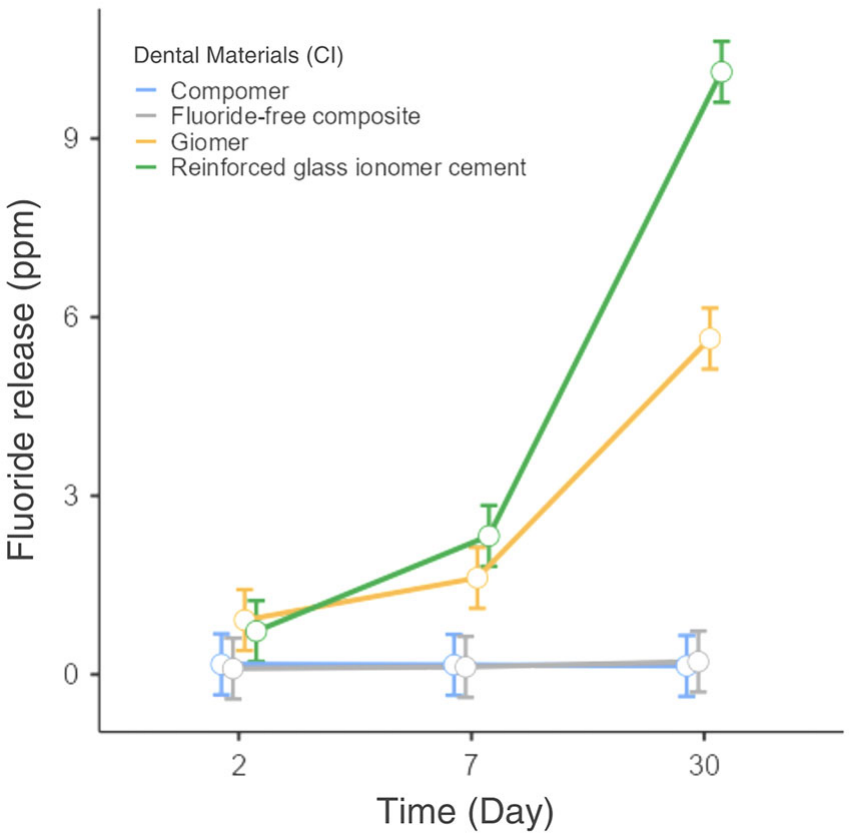
Fluoride release patterns according to confidence intervals (CI) for all dental materials tested over a 30‐day evaluation period.

Giomer exhibited a clear temporal release profile, with increases detected between days 2 and 30, and between days 7 and 30. These results support a cumulative release pattern, with fluoride release intensifying over time (Table 2 and Figure 2).

The resin‐reinforced glass ionomer also demonstrated consistent variation across all time points. A difference was observed between days 2 and 7, and a further increase between days 7 and 30, consistent with a progressive release trend. The comparison between days 2 and 30 revealed a markedly negative logarithmic difference, indicating that fluoride release at 2 days was virtually undetectable relative to the level observed at 30 days. Although mathematically valid, this extreme value likely reflects a delayed or initially undetectable release, followed by a pronounced cumulative effect. In contrast, compomer maintained relatively stable fluoride release throughout the 30‐day period (*p* > 0.05 for all comparisons), with no major fluctuations (Table 2 and Figure 2).

Direct comparisons between giomer and resin‐reinforced glass ionomer cement revealed no substantial differences at any time point (Table 3). Nonetheless, both materials displayed distinct release behavior compared with the other groups, especially from day 7 onward (Table 3 and Figure 2).

Figure 2 illustrates the fluoride release profiles (ppm) and corresponding 95% confidence intervals for all materials across the three time points. Fluoride‐free composite and compomer exhibited stable, low‐level release patterns, with minimal fluctuations. Giomer and resin‐reinforced glass ionomer, on the other hand, showed increasing release trends over time, with the most prominent changes occurring between days 7 and 30. Notably, the resin‐reinforced glass ionomer reached the highest absolute fluoride values by the end of the observation period. These findings align with the statistical results, which confirmed a material‐by‐time interaction effect, reinforcing that the dynamics of release varied between materials over time.

**FIGURE 3:**
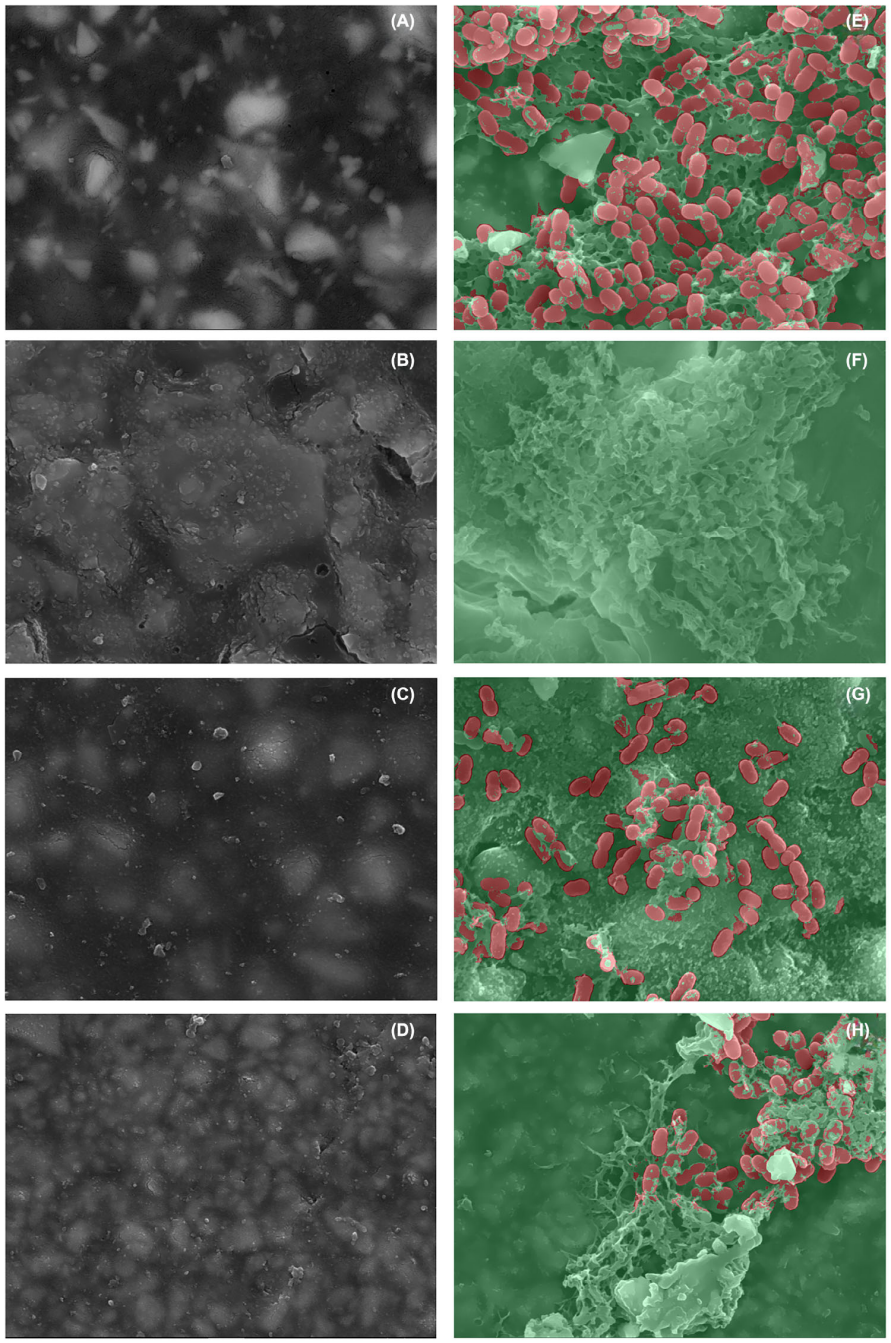
SEM analysis of the biofilm formed by *S. mutans* strain (ATCC25175) with an inoculum of 10^6^ CFU/mL, in BHI medium enriched with 2% sucrose. SEM images showing before and after 48 h of incubation, the quantitative analysis of *S. mutans* growth. Fluoride‐free composite/negative control: (A, E) reinforced glass ionomer cement: (B, F), giomer: (C, G), and compomer: (D, H). BHI, brain heart infusion; CFU, colony‐forming unit; SEM, scanning electron microscopy.

The SEM images obtained before and after 48 h of exposure to *S. mutans* revealed bacterial adhesion and extracellular polysaccharide production beneath the surface of all evaluated materials (Figure 3). Quantitative assessment was performed under double‐blind conditions. A visual subjective evaluation indicated greater bacterial presence on the fluoride‐free material. In the quantitative analysis, only colony‐forming units (CFUs), highlighted in red, were considered. The highest CFU count was recorded for the fluoride‐free material (190 CFUs), followed by giomer (72 CFUs), compomer (47 CFUs), and the resin‐reinforced glass ionomer, which exhibited no detectable CFUs.

## DISCUSSION

The principal finding of this in vitro study was that the resin‐modified glass ionomer and the giomer (S‐PRG‐based) sealants exhibited the highest fluoride release over the evaluation period, surpassing that of other restorative materials tested. However, this elevated fluoride release was not associated with a significant reduction in *S. mutans* biofilm adhesion.

A major strength of this study lies in its comprehensive approach to evaluating both the physicochemical properties (fluoride release) and biological effects (bacterial adhesion) of the materials under controlled conditions. The use of ion chromatography and SEM analysis allowed precise quantification and visual documentation. Nonetheless, the in vitro design represents a limitation, as it may not fully replicate the complex dynamics of the oral environment. Moreover, testing was restricted to a single bacterial species and conducted at a neutral pH, potentially underrepresenting the impact of more acidic and polymicrobial conditions typical of cariogenic biofilms.

In our previous study [22], we investigated the potential of fluoride‐releasing sealants in preventing enamel demineralization and inhibiting the metabolic activity of *S. mutans* within 48 h, highlighting their protective properties and underscoring the importance of fluoride as an antimicrobial agent. Fluoride is widely recognized for its ability to inhibit *S. mutans* enolase, disrupting acid production or growth pathways, which contributes to its potential anticariogenic effects [37]. Aware not only of the importance of fluoride in controlling demineralization processes but also of its potential interference in bacterial metabolism, Mayumi et al. [24] tested the action of dental materials with S‐PRG nanofiller technology (having fluoride as one of the released ions), using human dentin blocks. Their study found ion‐releasing properties, bacterial growth inhibition, and sterilization effects, concluding that S‐PRG nanofiller coating provides antibacterial protection to tooth surfaces. Conversely, the results of this study raised questions about the influence of time on fluoride release. In the present research, giomer (S‐PRG technology) and the resin‐modified glass ionomer sealant exhibited greater fluoride release than the other sealants, possibly due, respectively, to the presence of fluoride on their reactive surface, which stabilizes thereafter. This phenomenon may be attributed to the other sealants, for example, the compomers and resin‐based sealants, having a denser polymeric mesh that releases fluoride more slowly and in smaller quantities [38].

The discrepancy in the amount of fluoride released by the ionomer‐based and giomer materials compared with the other tested materials may be attributed to the controlled conditions of the in vitro study, which limits the extrapolation of results to real clinical scenarios, particularly in acidic pH environments. In contrast, Nicholson et al. [39] reported that fluoride release from glass ionomer materials occurs through a prolonged diffusion process under neutral pH conditions, which can reduce the size and viability of oral bacterial populations. In other studies, fluoride release has been associated with the remineralization or prevention of demineralization in underlying hard dental tissues, supporting the classification of resin‐modified glass ionomer sealants as bioactive restorative materials [22, 40, 41, 42, 43]. The present study also tested the compomer, which showed low fluoride release compared with the resin‐modified glass ionomer and giomer, with levels close to those of the negative control. This finding is consistent with the one described by Francois et al. [20] in a review of the limitations associated with fluoride release from resin materials.

Other factors related to bacterial adhesion emerge as important aspects for future research, such as the controversy surrounding the metabolic activity of *S. mutans* in the presence of fluoride [22, 55]. This underscores the relevance of not only evaluating fluoride release but also assessing its impact on biofilm viability and structural organization. This study focused on selected parameters feasible within the scope of the experimental design and available resources. Nonetheless, the inclusion of additional variables such as surface roughness, free surface energy, *S. mutans* metabolic activity at different time points, quantitative analysis of biofilm adhesion, and fluoride release under acidic conditions would considerably enhance the scientific robustness and clinical relevance of the findings. These characteristics should be taken into account, as they may influence microbial biofilm control on dental materials, including sealants.

The studies by Chau et al. [58] and Pandit et al. [59] support this broader context by providing valuable insights into the relationship between fluoride‐releasing materials and biofilm accumulation. Future investigations are encouraged to incorporate these complementary analyses and further explore the complex interactions between fluoride release and *S. mutans* biofilm dynamics.

The limitations of our study are primarily related to its in vitro nature, and therefore, the results should not be directly extrapolated to clinical settings. Additionally, only a single bacterial species was tested, as the biofilm adhesion evaluated was monomicrobial on the surface of dental materials. Furthermore, fluoride quantification was performed at only one pH level, which may not fully represent the dynamic conditions of the oral environment. These factors should be considered when interpreting the findings.

The observed results may be partially explained by differences in fluoride release mechanisms across materials. Giomers and glass ionomers release ions through more efficient surface or matrix diffusion, while compomers and resin composites exhibit delayed and lower ion release due to denser polymer networks. These findings emphasize that anticaries effect of fluoride is primarily physicochemical, acting during acidogenic episodes to modulate demineralization and to promote remineralization, rather than functioning as a potent antibacterial agent per se.

From a clinical perspective, these results imply that while fluoride‐releasing sealants can support caries prevention, they should not be solely relied upon for biofilm control. Additional material properties, such as lower surface roughness and optimal hydrophilicity, must be considered for reducing bacterial colonization.

This study leaves unanswered questions regarding the interplay between pH, biofilm composition, and long‐term fluoride release dynamics. Future research should investigate these variables under conditions that mimic clinical scenarios more closely, incorporating polymicrobial biofilms, cyclic pH fluctuations, and long‐term evaluations. In addition, studies should explore the integration of other bioactive agents that complement the physicochemical benefits of fluoride with enhanced antimicrobial action.

## AUTHOR CONTRIBUTIONS

**Conceptualization**: Pereira da Silva PSL. **Methodology**: Pereira da Silva PSL, Teixeira MS, de Araújo Rocha A, and Barros RR. **Resources**: Pereira da Silva PSL, de Araújo Rocha A, and Barros RR. **Data curation**: Pereira da Silva PSL. **Formal analysis**: Pereira da Silva PSL, Teixeira MS, de Araújo Rocha A, Gonçalves LS, and Tostes MA. **Writing—original draft**: Pereira da Silva PSL, de Araújo Rocha A, and Gonçalves LS. **Review and editing**: Pereira da Silva PSL, Gonçalves LS, and Tostes MA. **Project administration**: Tostes MA.

## CONFLICT OF INTEREST STATEMENT

The authors report no conflicts of interest with the participants or any other collaborators, whether direct or indirect, in relation to the development of this research.
